# Mechanical and Energy Evolution Characteristics of Fractured Sandstone Materials: A True Triaxial Experimental Study

**DOI:** 10.3390/ma18010175

**Published:** 2025-01-03

**Authors:** Guowen Sun, Yu Lu, Gun Huang, Qinming Liang, Xinyu Huang

**Affiliations:** 1 Technology Department, Chongqing Vocational Institute of Engineering, Chongqing 402260, China; 2 State Key Laboratory of Coal Mine Disasters Dynamics and Control, Chongqing University, Chongqing 400044, China; 3 School of Resources and Safety Engineering, Chongqing University, Chongqing 400044, China

**Keywords:** true triaxial stresses, fractured rock, crack initiation stress, crack damage stress, energy evolution

## Abstract

To investigate the mechanical and energy evolution characteristics of fractured rock under true triaxial stresses, true triaxial strength compression experiments on fractured sandstone were conducted with varying crack lengths and widths. The results indicate that under true triaxial stresses, the peak stress of the rock exhibits a gradual decline with an increase in crack length and width. Meanwhile, crack initiation stress and crack damage stress of fractured sandstone also demonstrate a declining trend overall, and the influence of crack length on the characteristic stress (crack initiation stress and crack damage stress) of sandstone is more pronounced than that of crack width. According to the energy analysis results, the total strain energy of fractured sandstone gradually decreases with an increase in crack length and width. The results offer a theoretical foundation for the strength assessment and stability management of fractured rock materials during deep coal mining operations.

## 1. Introduction

China is a major coal-producing country with abundant coal reserves, meaning that coal will remain the dominant energy source for a long time. Currently, shallow resources are becoming increasingly depleted, and coal resource extraction in China, particularly in the central and eastern regions represented by Northeast and East China, is advancing to deeper levels at a rate of 10–25 m/a, reaching depths of 800–1000 m [1]. As coal mining extends into deeper regions, the stress conditions of the surrounding rock become increasingly complex, with a large number of joints and fractures present. Studying the failure characteristics of rocks containing joints and fractures can provide technical support for deep coal mining and utilization, improving resource extraction efficiency and economic benefits [2].The length and width of fractures can significantly affect the strength and deformation capacity of rocks [3]. By studying the impact of fracture length and width on rock strength, the load-bearing capacity and deformation behavior of rocks can be more accurately assessed [4]. Since rocks, during deep mining, are often subjected to true triaxial stress conditions with unequal pressures in three directions, it is necessary to study the effects of fracture length and width on the mechanical properties and energy characteristics of rocks under true triaxial stresses conditions. This helps us better understand and predict the mechanical behavior of rocks and provides reference data for the design and construction of deep coal mining projects [5].

Uniaxial compression tests are widely used to study the strength and deformation characteristics of rocks, allowing the determination of parameters such as compressive strength, elastic modulus, and plastic deformation characteristics. Zhou et al. [6] reproduced the initiation, propagation, and failure processes of prefabricated fractures at various angles in rock-like specimens through indoor uniaxial compression experiments, validated by numerical simulations. Wang et al. [7] combined uniaxial compression tests and numerical simulations to find that pillar strength decreases with increasing fracture density, and rock strength exhibits a “U-shaped” variation with increasing fracture inclination. Guo et al. [8] studied the mechanical behavior of layered fractured red sandstone specimens with different fracture lengths and widths through uniaxial compression and Brazilian tests. Zhao et al. [9] demonstrated through biaxial compression tests of fractured rocks that the initial stress, peak strength, and the number of shear cracks under biaxial compression are significantly greater than those under uniaxial compression. Han et al. [10] conducted biaxial compression tests on fractured rock specimens and found that the angle between the fractures and the principal stress direction significantly affects the peak strength of the specimens, with the lowest strength at a 30° angle and the highest at a 90° angle. In conventional triaxial compression tests, Takahashi et al. [11] found that the failure strength of specimens depends primarily on the angle between the joint fractures and the maximum principal stress. Specimens with sliding failure along the joint surface exhibit significantly lower strength compared to intact rocks. Wang et al. [12] performed triaxial compression and creep tests on sandstone specimens with a single fracture, revealing that the steady-state creep rate of fractured sandstone is higher than that of intact specimens, and the creep rate first increases and then decreases with increasing fracture angle. Yang et al. [13] showed, in triaxial compression tests of fractured rocks, that prefabricated fractures significantly affect the stress–strain curve, with more stress drops observed before and after the peak strength, and confining pressure not reducing these stress drops. The strength and deformation characteristics of rocks also directly influence their energy evolution characteristics, prompting researchers to study the energy features of fractured rocks. Zhang et al. [14] demonstrated that the energy storage limit of rock specimens under triaxial loading is higher than that under uniaxial compression or triaxial unloading. Gong et al. [15] revealed that under triaxial loading and unloading, the failure mode of fractured granite is primarily dominated by shear failure, and the energy storage limit decreases initially and then increases with increasing fracture inclination. Majedi et al. [16] did well in modelling the static behavior of sandstone specimens by numerical simulation results, and further introduced micromechanical models to reproduce the physical experimental data in the related literature. Afrazi et al. [17] investigated the critical stresses of shear cracking series by way of the digital image correlation technique and numerical simulation to investigate the material grain size relationship. Li et al. [18] found, through uniaxial compression of fractured rocks, that as the distance between a horizontal single fracture and the specimen ends decreases, the strain energy of the rock also decreases, reflecting the damage and failure process of fractured sandstone through its energy evolution characteristics.

In summary, extensive research has been conducted on the effects of different fracture angles on the mechanical properties of rocks, uniaxial and biaxial compression tests, and conventional triaxial tests of prefabricated fractured rocks [19], as well as their mechanical characteristics and energy evolution. However, research on prefabricated fractured specimens under true triaxial conditions has mainly focused on the mechanical characteristics under the minimum principal stress, with limited studies on the fracture characteristics of sandstone in the intermediate principal stress direction. Additionally, research on the mechanical characteristics and energy evolution of fracture length and width is scarce [20]. Therefore, studying the effects of fracture length and width on the mechanical properties and energy evolution of rocks in the intermediate principal stress direction under true triaxial conditions is essential. It deepens our understanding of the mechanical behavior of surrounding rock during deep mining, predicts failure mechanisms, and optimizes engineering design. Using a self-developed true triaxial fluid–solid coupling test system, triaxial strength tests were conducted on sandstone specimens with different fracture lengths and widths. The characteristic stresses (initiation stress, damage stress, and peak stress) of the specimens were investigated under various conditions, and the elastic modulus was calculated [21], along with an analysis of energy evolution characteristics.

## 2. Materials and Methods

### 2.1. Experimental Equipment

**Figure 1:**
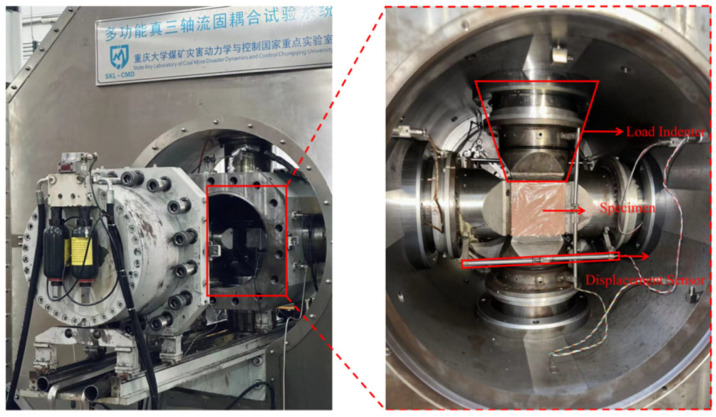
True triaxial fluid–solid coupling test system.

This experiment was conducted using the multifunctional true triaxial fluid–solid coupling test system developed by the National Key Laboratory of Coal Mine Disaster Dynamics and Control at Chongqing University [22], as shown in Figure 1. The multifunctional true triaxial fluid–solid coupling test system primarily consists of a frame structure, a true triaxial pressure chamber, a loading system, an internal sealed seepage system, a control and data measurement and acquisition system, and an acoustic emission monitoring system [23].

### 2.2. Specimen Preparation

**Figure 2:**
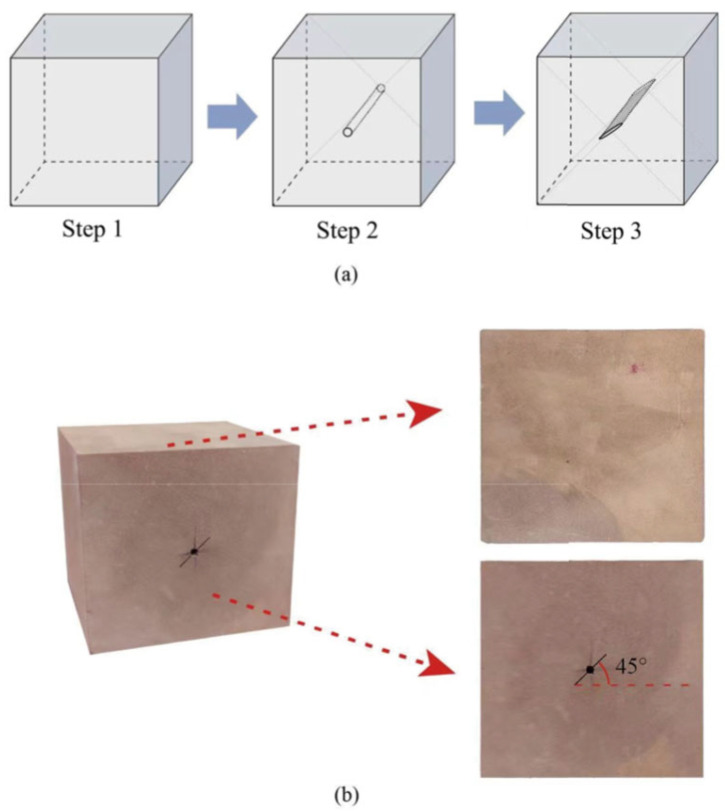
Specimen preparation. (**a**) Sample Processing Diagram. (**b**) Physical rock sample.

The test specimens were made of red sandstone, which is widely distributed in underground coal mining projects. Red sandstone has advantages such as uniform texture, high strength, and ease of processing [24]. The sandstone was sourced from the Qixin Coal Mine in Yaan, Sichuan Province, Southwest China. The rock specimen has a density of 2260 kg/m^3^, a uniaxial compressive strength of 41.6 MPa, an elastic modulus of 5.4 GPa, and a Poisson’s ratio of 0.36. The XRD analyses show that the mineral fraction of this sandstone consists of quartz (42%), feldspar (2.4%), clay minerals (51.6%), rhodochrosite (3.8%), and others (0.2%). All rock specimens used in the tests were taken from the same complete and homogeneous sandstone block to ensure uniformity in internal structure and properties. The sandstone blocks were cut and processed into cubic specimens measuring 100 mm × 100 mm × 100 mm. The surfaces of the specimens were polished to ensure smoothness and that they were free from significant defects, with flatness and perpendicularity deviations less than 0.02 mm. Artificial prefabricated fractures were created using a central drilling method and a diamond wire cutter to generate the fractures. The fracture inclination angle was set at 45°, and the prefabricated fracture parameters included fracture inclination, width, length, and connectivity, as shown in Figure 2. For specimens with varying fracture lengths, the parameters were as follows: fracture inclination of 45°, width of 0.5 mm, and lengths of 10 mm, 15 mm, 20 mm, and 25 mm. For specimens with varying fracture widths, the parameters were as follows: fracture inclination of 45°, length of 20 mm, and widths of 0.5 mm, 0.7 mm, 0.9 mm, and 1.1 mm. The specimen parameters are summarized in Table 1.

### 2.3. Stress Loading Procedure

**Figure 3:**
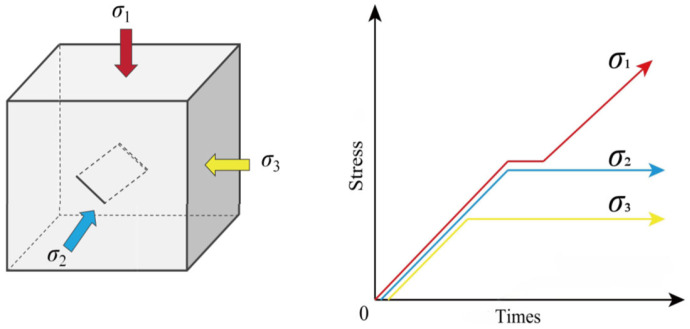
Schematic of stress path setup.

1. Apply triaxial stress simultaneously using the force control mode (0.2 kN/s) until reaching hydrostatic stress conditions of *σ*_1_ = *σ*_2_ = *σ_3_* = 15 MPa.
2. Maintain the minimum principal stress *σ_3_* = 15 MPa constant, and use the force control mode (0.2 kN/s) to increase the intermediate principal stress σ_2_ and the maximum principal stress σ_1_ until *σ*_1_ = *σ*_2_ = 30 MPa.
3. Maintain the minimum principal stress *σ_3_* = 15 MPa and the intermediate principal stress *σ*_2_ = 30 MPa, then apply the maximum principal stress *σ*_1_ at a constant displacement rate of 0.001 mm/s until the specimen fails [25], as shown in Figure 3.

## 3. Experimental Results and Analysis

### 3.1. Stress–Strain Curve Analysis

**Figure 4:**
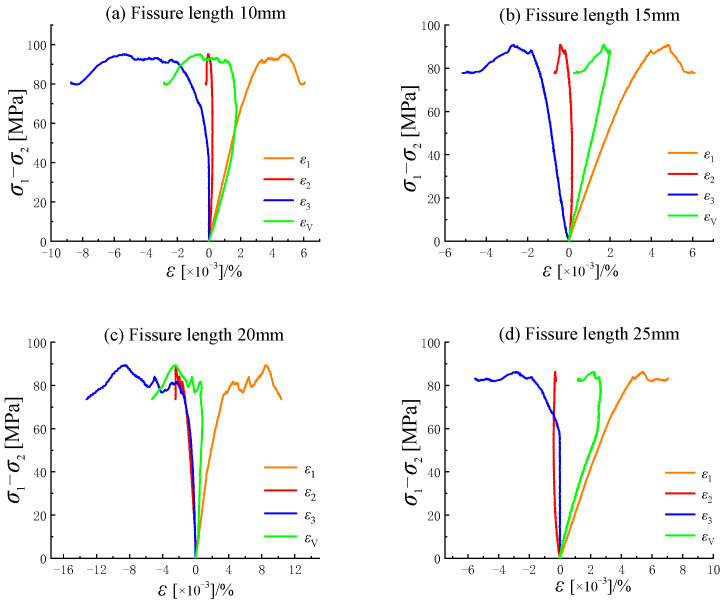
Stress–strain curves of sandstones with different fissure lengths under true triaxial stresses.

**Figure 5:**
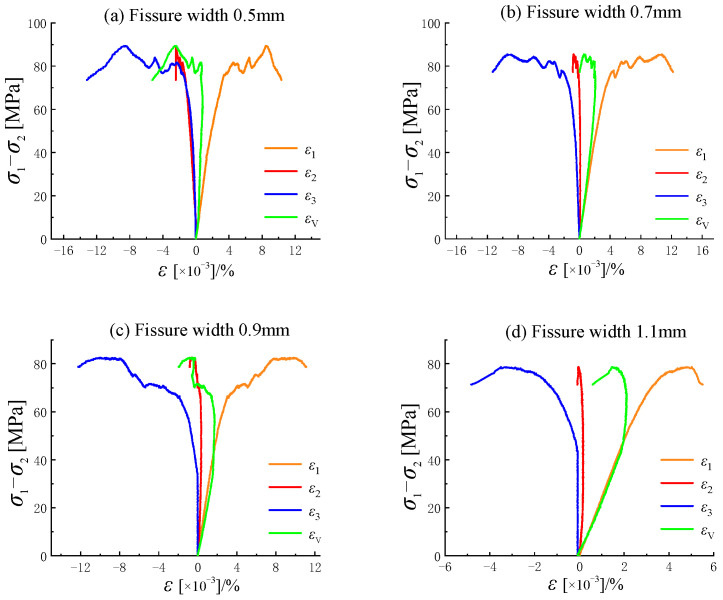
Stress–strain curves of sandstones with different fissure widths under true triaxial stresses.

The stress–strain curve exhibits an approximately linear increase during the initial loading stage. At this point, the load has not reached the yield strength required to close the fractures, so this stage is considered the elastic phase. As the load continues to increase, the internal cracks of the specimen close, and cracks initiate at the tips of the fractures. The curve transitions from linear to nonlinear, entering the yield phase. When cracks continue to develop, the rising trend of the stress–strain curve slows, and volumetric expansion occurs in the rock specimen. The volumetric strain of the crack transitions smoothly from compression to dilation. After compression along the direction of the maximum principal stress, the specimen undergoes compressive deformation, while dilation deformation occurs in the directions of the minimum and intermediate principal stresses due to volumetric expansion. The prefabricated fractures in the specimen gradually propagate to the corners or edges, causing through-going failure. After failure, the stress–strain curve drops rapidly, marking the failure phase. As shown in Figure 4 and Figure 5, the stress–strain curves of rock specimens with different fracture lengths at the same inclination angle exhibit similar trends [26]. The peak strength of the stress–strain curve decreases as fracture length increases. The intermediate principal strain shows a multi-stage increase when the fracture length is 20 mm, whereas it exhibits a single peak at other lengths. As shown in Figure 5, with increasing fracture width at a 45° inclination angle, specimens with widths of 0.5 mm, 0.7 mm, and 0.9 mm exhibit a distinct multi-stage increase in peak stress, particularly evident in the post-peak failure phase, where the curve drops steeply. This indicates significant ductile failure characteristics in specimens with fractures. At a fracture width of 1.1 mm, the peak curve rises smoothly, showing a single peak. In all cases, the stress–strain curves of fractured rocks exhibit an evident stress drop after the peak, indicating pronounced brittle failure characteristics.

### 3.2. Determination of Rock Initiation and Damage Stresses

The crack volumetric strain method is one of the most widely used methods for determining characteristic stresses [21]. Therefore, this paper adopts the crack volumetric strain method to study the crack initiation stress and damage stress of sandstone with different fracture lengths and widths.

The crack volume strain $\varepsilon_{v}$ consists of the strain $\varepsilon_{1}$ in the direction of the maximum principal stress $\sigma_{1}$, the strain $\varepsilon_{2}$ in the direction of the intermediate principal stress $\sigma_{2}$ and the strain $\varepsilon_{3}$ in the direction of the minimum principal stress $\sigma_{3}$ in the true triaxial stress state, i.e.:

(1) $$\varepsilon_{v}=\varepsilon_{1}+\varepsilon_{2}+\varepsilon_{3}$$

(2) $$\varepsilon_{v}=\varepsilon_{ev}+\varepsilon_{cv}$$

According to the generalized Hooke’s law, the elastic volumetric strain *ε_ev_* equation for the true triaxial compression condition is obtained as:

(3) $$\varepsilon_{ev}=\frac{1-2\mu }{E}(\sigma_{1}+\sigma_{2}+\sigma_{3})$$

*μ* is Poisson’s ratio, which is taken as 0.24, and *E* is the elastic modulus, which is determined according to the elastic phase of the stress–strain curve.

From the combination of Equations (2) and (3), the crack volume strain *ε_cv_* is derived as:

(4) $$\varepsilon_{cv}=\varepsilon_{v}-\varepsilon_{ev}=\varepsilon_{1}+\varepsilon_{2}+\varepsilon_{3}-\frac{1-2\mu }{E}\left(\sigma_{1},+,\sigma_{2},+,\sigma_{3}\right)$$

**Figure 6:**
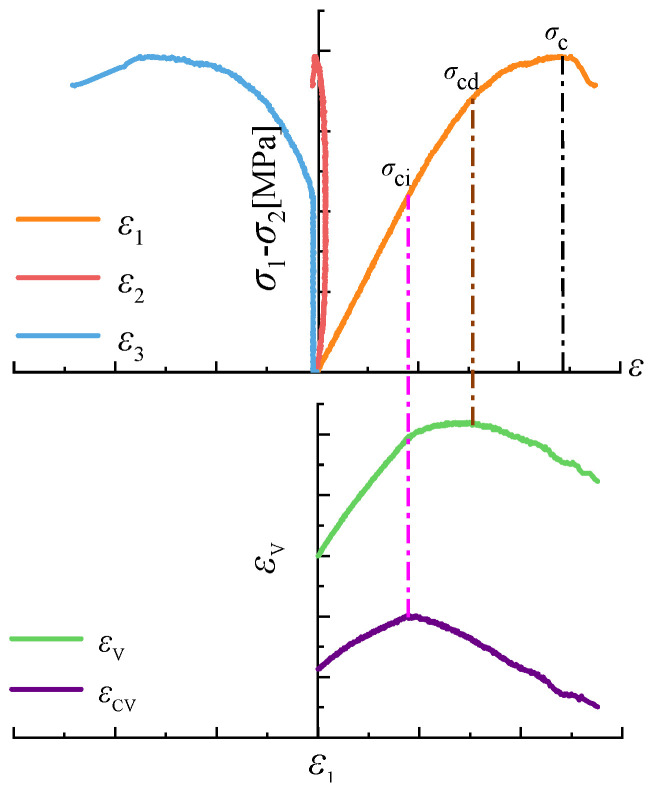
Characteristic stress determination.

The schematic diagram for determining the characteristic stresses of rock specimens is shown in Figure 6. Crack volumetric strain represents the volume reduction associated with the closure of initial cracks. When the rock specimen enters the crack propagation stage, the total volumetric strain includes the volume increase caused by crack propagation. As a result, the increase in total volumetric strain becomes smaller than the increase in elastic volumetric strain, causing the crack volumetric strain curve to slope in the negative direction. The inflection point on this curve corresponds to the crack initiation stress $\sigma_{ci}$, which represents the stress at which cracks begin to form in the rock. As the load continues to increase, the volumetric deformation of the rock transitions from compression to dilation, and the volumetric strain curve exhibits another inflection point. The axial stress level corresponding to this point is the damage stress [27]. This is the stress $\sigma_{cd}$ at which significant crack damage occurs in the rock and corresponds to the peak stress $\sigma_{c}$ of the rock. Under true triaxial compression conditions, the stress–strain curve of the rock is divided into four stages. After determining the elastic modulus during the elastic deformation stage, the curve transitions to the crack propagation stage, during which internal cracks in the rock begin to form and develop. The endpoint of this stage corresponds to the crack initiation stress of the rock, where the maximum value of the crack volumetric strain indicates the crack initiation stress. Based on varying stress levels and damage conditions, the crack propagation stage is further divided into a stable crack propagation stage and an unstable crack propagation stage. The damage stress represents the boundary between these two stages. When the volumetric strain reaches its maximum value, the corresponding stress is the rock’s damage stress $\sigma_{cd}$. The peak stress $\sigma_{c}$ indicates the rock’s ultimate strength. Upon further loading, the internal cracks in the rock continue to propagate, eventually leading to macroscopic brittle failure. At this point, the stress–strain curve shows a steep post-peak decline [28].

### 3.3. Analysis of Crack Initiation and Damage Stress of Sandstone with Different Crack Lengths

**Figure 7:**
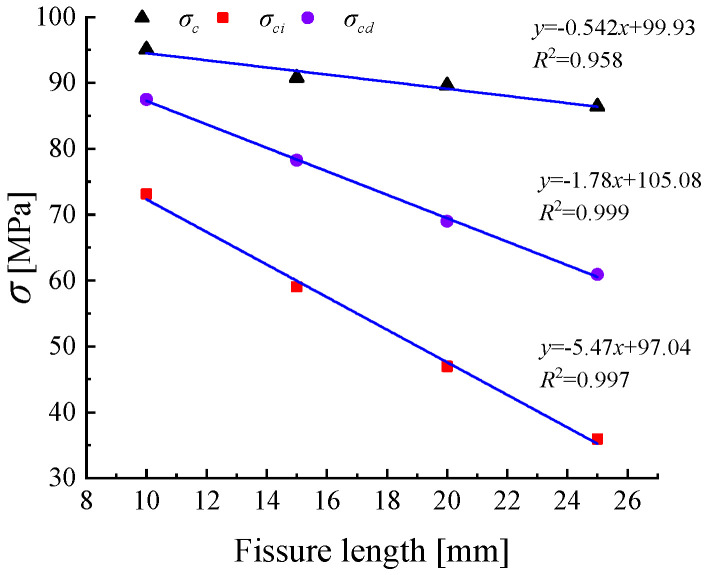
Characteristic stresses in rocks with different fissure lengths.

As the crack length increases, the crack initiation stress, damage stress, and post-peak failure of the specimens show varying degrees of difference. In Table 2, the crack initiation stresses $\sigma_{ci}$ for the four groups of sandstone specimens with crack lengths ranging from 10 mm to 25 mm are 73.15 MPa, 59.02 MPa, 46.95 MPa, and 35.96 MPa, respectively; the damage stresses $\sigma_{cd}$ are 87.50 MPa, 78.25 MPa, 68.98 MPa, and 60.90 MPa. As shown in Figure 7, with the increase in crack length, the rate of crack initiation and propagation accelerates, resulting in a continuous decrease in both the crack initiation stress and damage stress. The ratios of crack initiation stress and damage stress also follow this trend. Under the condition of a 10 mm crack length, the specimen requires the largest external load for crack closure, and the difference between damage stress and peak stress is relatively small. According to Hooke’s Law, the strain corresponding to the cracked specimen increases under compression, so as the crack length increases, the decreasing trends of crack initiation stress and damage stress overall decelerate in the cracking and compaction process. The peak strength overall shows a decreasing trend with increasing crack length, exhibiting a negative correlation. Figure 7 indicates that with the continuous increase in crack length, the peak strength of the rock decreases, and the difference between crack initiation stress and damage stress becomes more significant. The larger the macrocrack-to-overall-volume ratio of the rock, the greater the influence on the overall strength of the rock. This is consistent with the findings of Zhang Yan et al. [29].

Under the condition that the crack inclination angle is controlled at 45°, the peak strengths for crack lengths of 10 mm, 15 mm, 20 mm, and 25 mm are 95.04 MPa, 90.75 MPa, 89.59 MPa, and 86.40 MPa, respectively. The peak strength shows a gradual decrease as the degree of damage to the sandstone increases. When the crack length increases from 10 mm to 15 mm, there is a significant drop in the peak stress of 4.29 MPa. In the range of 15 mm to 20 mm, the decrease in peak stress is smaller, which is consistent with the findings of Wang et al. [30]. As seen in Table 2, sandstone specimens with larger elastic moduli exhibit an increase in crack initiation stress and damage stress, as sandstone has stronger deformation resistance during the elastic phase.

### 3.4. Analysis of Crack Initiation and Damage Stress of Sandstone with Different Crack Widths

As shown in Table 3, the crack initiation stress ratios for specimens with crack widths of 0.5 mm, 0.7 mm, 0.9 mm, and 1.1 mm are 56.12%, 51.07%, 43.68%, and 44.50%, respectively, while the damage stress ratios are 81.50%, 80.12%, 74.00%, and 70.42%. Sandstone specimens with larger elastic moduli show an increase in crack initiation stress and damage stress, indicating that sandstone has a stronger deformation resistance during the elastic phase. This result is consistent with the findings for crack length, where elastic modulus also affects crack initiation and damage stresses.

As the crack width increases, the existing crack is more prone to deformation under stress. Table 3 shows that when the crack width increases from 0.7 mm to 1.1 mm, the elastic modulus of the specimen decreases. When the crack length is fixed at 20 mm and the inclination angle is 45°, the mechanical characteristics exhibited by crack widths of 0.9 mm and 1.1 mm are very similar. This suggests that while the crack width does affect the mechanical strength of sandstone, its impact on crack initiation stress is limited.

**Figure 8:**
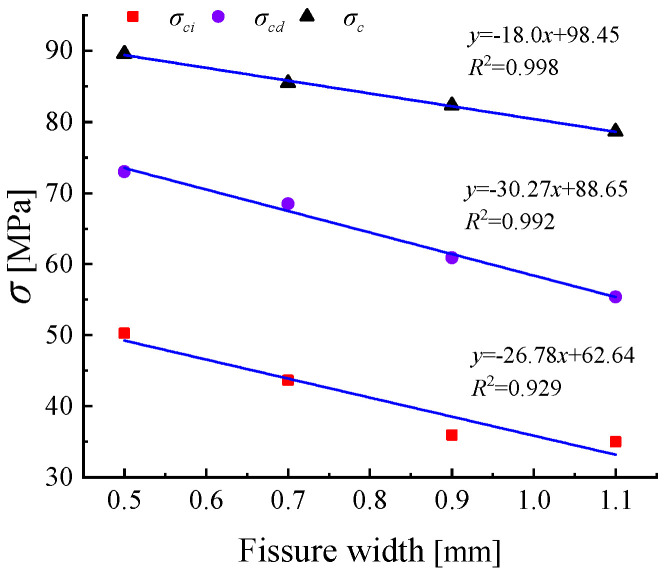
Characteristic stresses of rock specimens with different fissure widths.

When the crack inclination angle is 45°, the peak strengths for crack widths of 0.5 mm, 0.7 mm, 0.9 mm, and 1.1 mm are 89.59 MPa, 85.47 MPa, 82.32 MPa, and 78.63 MPa, respectively. As shown in Figure 8, as the crack width gradually increases, the peak strength of the specimen gradually decreases. When the crack width is increased by 0.2 mm in steps, the peak strength of the specimen shows a nearly consistent downward trend, with a decrease in strength ranging from 3.15 MPa to 4.12 MPa. The maximum difference in the downward trend between successive increments is only 0.97 MPa. The peak strength of the specimen decreases by an average of 3.65 MPa for every 0.2 mm increase in crack width. Between crack widths of 0.5 mm and 1.1 mm, when the increment of crack width is fixed, the reduction in rock specimen strength is nearly consistent.

The trends of crack initiation stress $\sigma_{ci}$ and damage stress $\sigma_{cd}$ are shown in Figure 8. The changes in crack initiation stress and damage stress of the specimens with increasing crack width exhibit a similar trend to the changes in crack initiation stress and damage stress with increasing crack length. As the proportion of the crack volume to the total specimen volume gradually increases, the specimen reaches the stages of crack closure, crack initiation, and propagation later under true triaxial stress conditions. During compression, the corresponding strain of the cracked specimen increases, and the overall cracking and compaction process slows down [31]. However, the effect of crack width on the crack initiation stress and damage stress of sandstone is not as significant as the effect of crack length.

## 4. Analysis of the Energy Evolution of Fractured Sandstones Under True Triaxial Stresses

Under the condition of true triaxial stress, four main energy conversion processes occur in rock: energy input, energy accumulation, energy dissipation and energy release. The total strain energy of the rock during the true triaxial compression test is *U*, which is known from the first law of thermodynamics:

(5) $$U=U_{d}+U_{e}+U_{0}$$

*U* is the total strain energy; *U_e_* is the elastic strain energy; *U_d_* is the dissipated energy; and *U*_0_ is the energy released by thermal radiation, heat exchange, etc. The test process is assumed to be a closed device, so *U*_0_ is neglected. The true triaxial loading equipment loading process tends to be static loading, so the kinetic energy is negligible.

(6) $$U=\int_{0}^{\varepsilon_{1}}\sigma_{1}d\varepsilon_{1}+\int_{0}^{\varepsilon_{2}}\sigma_{2}d\varepsilon_{2}+\int_{0}^{\varepsilon_{3}}\sigma_{3}d\varepsilon_{3}$$

(7) $$U_{e}=\frac{1}{2}(\sigma_{1}\varepsilon_{1}^{e}+\sigma_{2}\varepsilon_{2}^{e}+\sigma_{3}\varepsilon_{3}^{e})$$

$\varepsilon_{i}^{e}$ is the elastic strain in each direction, $\;and\;\varepsilon_{i}$ is the strain in each direction.

**Figure 9:**
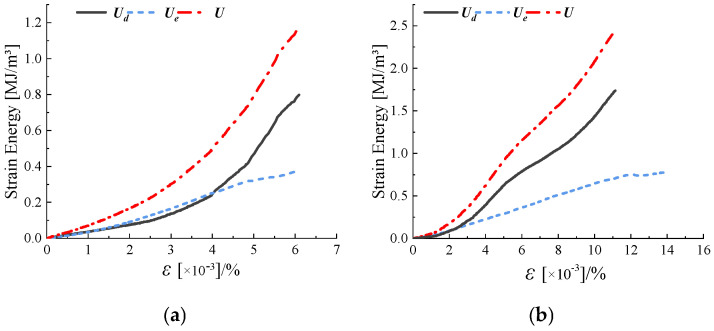
Typical energy curves for sandstones with different fissure geometries ((**a**) is typical energy for different fissure lengths; (**b**) is typical energy for different fissure widths).

Figure 9 shows the typical energy curves of sandstone with different fracture lengths (a) and different fracture widths (b) under true triaxial stress. The strain energy of the rock is positively correlated with its strain. The peak point of the elastic strain energy curve represents the energy storage limit, reflecting the rock’s ability to resist failure. Energy dissipation affects rock failure by initiating microcracks that develop into macroscopic cracks until the rock ultimately fails. During the elastic deformation phase and the microcrack development phase, the dissipated energy of the rock remains relatively low, and the total strain energy is primarily provided by the elastic strain energy. At this stage, the bonding energy of the rock’s microscopic particles exceeds the strain energy. Regardless of the sandstone’s fracture width or length, as it enters the crack propagation phase, and the total strain energy curve and the elastic strain energy curve gradually diverge. Simultaneously, the dissipated energy strain curve increases sharply with strain. A significant amount of energy dissipates during the development of microcracks, where the bonding energy of the rock’s microscopic particles becomes less than the strain energy. The dissipated energy manifests as the accumulation of rock damage and plastic deformation. When the elastic strain energy curve reaches its peak and the dissipated strain energy curve rises sharply, it indicates the development and propagation of macroscopic cracks, leading to final failure. At this stage, dissipated energy dominates the total strain energy.

### 4.1. Energy Evolution Characteristics of Sandstone with Different Fracture Lengths and Widths

**Figure 10:**
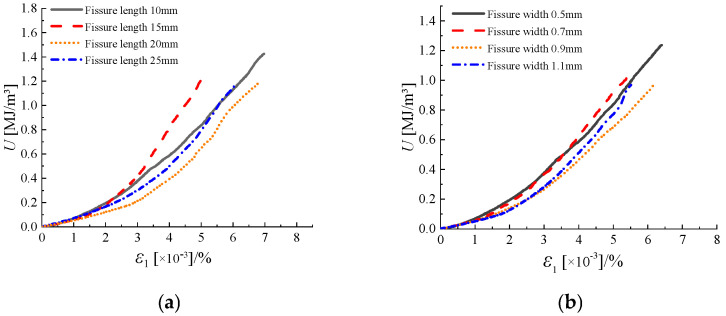
Total strain energy curvature of sandstone with different fissure geometries ((**a**) is the total strain energy of sandstone with different fissure lengths; (**b**) is the total strain energy of sandstone with different fissure widths).

As shown in Figure 10, the total strain energy of sandstone decreases with increasing fracture length. This indicates that the longer the geometric length of the joints and fractures in sandstone, the greater the degree of damage to the sandstone. The time required for the rock to enter the crack propagation phase is shortened, and the energy required for final failure decreases. The longer the fracture length, the lower the rock’s energy storage limit. The significant energy dissipation for sandstone with different fracture lengths primarily occurs after the peak of the stress–strain curve, and this phenomenon is not influenced by the geometric length of the fractures.

Similarly, the total strain energy of sandstone decreases with increasing fracture width. The greater the geometric width of the fractures, the higher the degree of damage to the sandstone, resulting in lower peak elastic strain energy and reduced resistance to failure. The total strain energy curves for sandstone with varying fracture widths exhibit less divergence compared to those with varying fracture lengths, indicating that fracture width has a smaller effect on the energy dissipation phase than fracture length.

### 4.2. Analysis of Rock Strength and Energy Evolution

**Figure 11:**
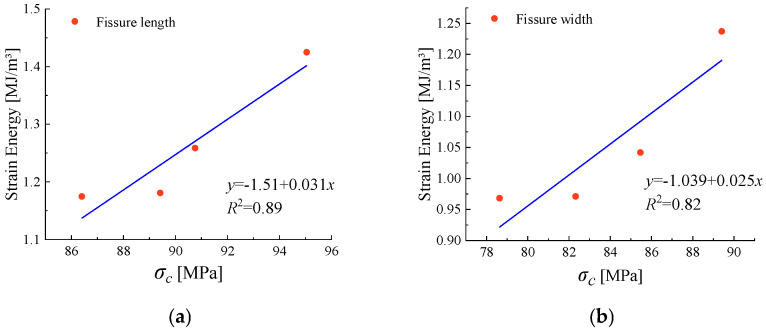
Trend of total strain energy with peak stress ((**a**) is the trend of energy with stress for sandstones with different fracture lengths; (**b**) is the trend of energy with stress for sandstones with different fracture widths).

Figure 11 shows that the geometric dimensions of rock fractures, whether length or width, significantly weaken the original energy storage capacity of rocks under true triaxial stress, with evident energy dissipation. The specific energy changes are shown in Table 4. In the early stages of true triaxial fracturing, the energy dissipation curves of sandstone with different fracture lengths and widths tend to overlap, diverging at different crack initiation stresses. As seen from the Figure 11, as the fracture length and width of the sandstone decrease, the peak stress at which sandstone fails under true triaxial stress increases, the energy storage limit becomes higher, and the total strain energy consumed during failure also increases. Among the specimens, sandstone with a fracture length of 10 mm and a fracture width of 0.5 mm requires the most energy. As the fracture length and width increase, their impact on the required energy gradually diminishes. The required energy for fracture lengths of 20 mm and 25 mm and fracture widths of 0.9 mm and 1.1 mm is nearly identical, indicating that when the degree of rock damage exceeds a certain range, the weakening effect on the rock’s energy storage limit and resistance to failure reaches an upper limit.

## 5. Conclusions

(1) Under true triaxial stress, the peak strength of sandstone decreases with the increase in fracture length and width. Similarly, the initiation stress and damage stress of fractured sandstone decrease as fracture length and width increase. Fractures act as prefabricated defects in sandstone specimens, and their increasing scale (length and width) reduces rock strength, accelerating the sandstone’s progression to the stages of fracture closure, crack initiation, and propagation.
(2) As fracture width increases incrementally by 0.2 mm, the decrease in specimen strength exhibits a relatively clear pattern. However, the reduction in strength with increasing fracture length is less regular. The largest drop in peak strength occurs when fracture length increases from 10 mm to 15 mm. Fracture length has a stronger influence on the initiation stress and damage stress of sandstone than fracture width.
(3) The total strain energy of sandstone decreases with the enlargement of internal fracture geometric dimensions. Significant energy dissipation occurs after the peak stress. The impact of fracture width on the energy storage limit of sandstone is less significant than that of fracture length.
(4) Under true triaxial stresses, as fracture length and width increase, the damage to sandstone becomes more severe, the energy storage limit decreases, and the resistance to failure weakens. When the extent of damage to sandstone exceeds a certain threshold, the influence of fractures on resistance to failure and the energy storage limit under true triaxial stress reaches an upper limit.

Possible directions for future research: consider the effects of different environmental factors (e.g., temperature, humidity, chemical action) on the strength and damage characteristics of fissured sandstone; use a combination of numerical simulation and experimental methods to further study the damage mechanism of fissured sandstone under different stress paths; explore the stability of fissured sandstone in practical engineering, such as the progressive deformation evolution characteristics of the surrounding rock after excavation of the deep mine tunnel and its progressive failure mechanism.

## Figures and Tables

**Table 1:** Rock specimen parameters.

Specimens	Fissure Length [mm]	Fissure Width [mm]	*σ*_2_ [MPa]	*σ*_3_ [MPa]
Sandstone 1	20	0.5	15	30
Sandstone 2	20	0.7	15	30
Sandstone 3	20	0.9	15	30
Sandstone 4	20	1.1	15	30
Sandstone 5	10	0.5	15	30
Sandstone 6	15	0.5	15	30
Sandstone 7	20	0.5	15	30
Sandstone 8	25	0.5	15	30

**Table 2:** Characteristic stresses of specimens with different crack lengths.

Fissure Length [mm]	$\sigma_{ci}$ [MPa]	$\sigma_{cd}$ [MPa]	$\sigma_{c}$ [MPa]	$\sigma_{ci}/\sigma_{c}$	$\sigma_{cd}/\sigma_{c}$	*E* [GPa]
10	73.15	87.50	95.04	76.97%	92.07%	31.23
15	59.02	78.25	90.75	65.04%	86.23%	29.41
20	46.95	68.98	89.59	52.41%	77.00%	23.88
25	35.96	60.90	86.40	41.62%	70.49%	18.27

**Table 3:** Stresses on specimens with different crack widths.

Fissure Width [mm]	$\sigma_{ci}$ [MPa]	$\sigma_{cd}$ [MPa]	$\sigma_{c}$ [MPa]	$\sigma_{ci}/\sigma_{c}$	$\sigma_{cd}/\sigma_{c}$	*E* [GPa]
0.5	50.28	73.02	89.59	56.12%	81.50%	29.41
0.7	43.65	68.48	85.47	51.07%	80.12%	23.16
0.9	35.96	60.90	82.32	43.68%	74.00%	20.85
1.1	34.99	55.37	78.63	44.50%	70.42%	19.87

**Table 4:** Strain energy for different fracture sizes.

	Fissure Length [mm]	$\sigma_{c}$ [MPa]	Strain Energy [MJ/m^3^]	Fissure Width [mm]	$\sigma_{c}$ [MPa]	Strain Energy [MJ/m^3^]
	10	95.04	1.425	0.5	89.59	1.237
	15	90.75	1.256	0.7	85.47	1.041
	20	89.59	1.180	0.9	82.32	0.971
	25	86.40	1.174	1.1	78.63	0.968
maximum		95.04	1.425		89.59	1.237
minimum		86.40	1.174		78.63	0.968
average		90.445	1.259		84.01	1.054

## Data Availability

The original contributions presented in the study are included in the article, further inquiries can be directed to the corresponding author.

## Nomenclature

*σ*	stress
*σ*_1_	maximum principal stress
*σ*_2_	intermediate principal stress
*σ*_3_	minimum principal stress
*σ_c_*	peak stress
*σ_ci_*	cracking stress
*σ_cd_*	damage stress
*ε*	strains
*ε*_1_	maximum principal strain
*ε*_2_	intermediate principal strain
*ε*_3_	minimum principal strain
*ε_v_*	volumetric strain
*ε_cv_*	crack volume strain
*ε_ev_*	elastic volumetric strain
*E*	modulus of elasticity
*μ*	Poisson’s ratio
*U*	total strain energy
*U_d_*	dissipative energy
*U_e_*	elastic strain energy

## References

[1] Yuan L. (2021). Progress in deep mining response and disaster prevention and control. J. Coal Sci..

[2] Chen J.M., Ren Q.W., Tu Y.C., Wang X.S., Wu J.X., Gao P.J., Meng Y.C., Xu M.L., Huang W., Zhang Z.S. (2023). Experimental research and theoretical application of petrophysics based on pore-fracture theory. Georeview.

[3] Sun H., Chen S., Jin A., Zhu D. (2022). Uniaxial compressive strength characteristics and crack evolution law of fracture-containing rock specimens. J. Northeast. Univ. (Nat. Sci. Ed.).

[4] Xia Z., Zhang X., Yao C., Chen H., Yang J., Jiang Q., Zhou C. (2019). Experiments on seepage characteristics of post-peak ruptured rocks during loading and unloading of peri-compression. J. Coal.

[5] Luo D., Lu S., Su G., Tao H. (2023). Experimental study of true triaxial single-face airborne rockburst of granite containing prefabricated single fissure. Geotechnics.

[6] Zhou J., Wang K., Zhou W., Yao Y., Xie T. (2024). Uniaxial Compressive Damage Characteristics of Rock-like Materials with Prefabricated Conjugate Cracks. Appl. Sci..

[7] Wang B., Yao C., Yang J., Jiang S. (2018). Numerical Simulation of Macro-Meso Mechanical Behaviours of Sandstone Containing a Single Open Fissure under Uniaxial Compression. Eur. J. Environ. Civ. Eng..

[8] Guo W., Yu F., Tan Y., Zhao T. (2019). Experimental Study on the Failure Mechanism of Layer-Crack Structure. Energy Sci. Eng..

[9] Zhao C., Niu J., Zhang Q., Yu S., Morita C. (2019). Numerical Simulations on Cracking Behavior of Rock-Like Specimens with Single Flaws under Conditions of Uniaxial and Biaxial Compressions. J. Mater. Civ. Eng..

[10] Han G., Jing H., Jiang Y., Liu R., Su H., Wu J. (2018). The Effect of Joint Dip Angle on the Mechanical Behavior of Infilled Jointed Rock Masses under Uniaxial and Biaxial Compressions. Processes.

[11] Takahashi N., Takahashi M., Park H., Fujii Y., Takemura T., Kwasniewski M., Li X., Takahashi M. (2012). Deformation and Strength Characteristics of Kimachi Sandstone under Confined Compression and Extension Test Conditions. True Triaxial Testing of Rocks.

[12] Wang B., Li T., Zhu Q., Ran J., Du Y., Zhang H. (2023). Study on the Creep Properties and Crack Propagation Behavior of Single-Fissure Sandstone Based on the Damage Bond Model. Theor. Appl. Fract. Mech..

[13] Yang S.-Q., Tian W.-L., Liu X.-R., Huang Y.-H., Yang J. (2021). An Experimental Study on Failure Mechanical Behavior and Cracking Mechanism of Rectangular Solid Sandstone Containing Two Non-Coplanar Fissures under Conventional Triaxial Compression. Theor. Appl. Fract. Mech..

[14] Zhang L., Cong Y., Meng F., Wang Z., Zhang P., Gao S. (2021). Energy Evolution Analysis and Failure Criteria for Rock under Different Stress Paths. Acta Geotech..

[15] Gong H., Luo Y., Xu K., Wang X., Tao Y., Li X. (2023). Mechanical Characteristics of Failure and Rockburst Proneness of Fractured Granite from Shuangjiangkou Hydropower Station under Triaxial Loading and Unloading. Bull. Eng. Geol. Environ..

[16] Majedi M.R., Afrazi M., Fakhimi A. (2021). A Micromechanical Model for Simulation of Rock Failure Under High Strain Rate Loading. Int. J. Civ. Eng..

[17] Afrazi M., Lin Q., Fakhimi A. (2022). Physical and Numerical Evaluation of Mode II Fracture of Quasi-Brittle Materials. Int. J. Civ. Eng..

[18] Li K.-S., Chen L.-X., Zhao Z., Liu C.-X. (2023). Experimental Investigation on Mechanical, Acoustic, and Fracture Behaviors and the Energy Evolution of Sandstone Containing Non-Penetrating Horizontal Fissures. Theor. Appl. Fract. Mech..

[19] Liu H. (2021). Numerical simulation of rock fracture parameters under different fracturing conditions. Energy Technol. Manag..

[20] Zhao G., Liu Z., Meng X., Zhang R., Gu Q., Qi M. (2023). Energy evolution of sandstone under true triaxial cyclic principal stress. Geotechnics.

[21] Li C., Xie H., Xie L. (2017). Experiment and theory of shale initiation stress and crack damage stress. J. Coal.

[22] Yin G.C., Ma B., Liu C., Li M.H., Lu J., Yin S.Y. (2019). Effects of loading and unloading rates on mechanical properties and energy characteristics of sandstone under true triaxial stress conditions. J. Coal.

[23] Yin G.C., Li M.H., Xu J., Wang W.Z., Li W.P., Li S., Song Z.L., Deng B.C. (2015). Development and application of a multifunctional true three-axis fluid-solid coupling test system. J. Rock Mech. Eng..

[24] Zheng X., Wang Y., Song M., Karim S.I. (2024). Strength Characteristics of Different Stress Spaces of Red Sandstone under Loading and Unloading Tests. Sci. Rep..

[25] Wang Z., Shi W., Kong R., Guo J. (2023). Mechanical Characterization of Deep Sandstone under True Three-Way Stress. J. Northeast. Univ. (Nat. Sci. Ed.).

[26] Liu H.N., Wang J.M., Wang S.J. (2010). Experimental study on true triaxial modeling of columnar jointed rocks in Baihetan. Geotech. Mech..

[27] Zhao J., Guo G., Xu D., Huang X., Hu Y.-Y., Xia Y.-L., Zhang D. (2020). Experimental characterization of deformation and damage of deeply buried hard rock under triaxial and cyclic loading and unloading stress paths. Geotechnics.

[28] Li B., Gong J., Long Y., Hu H., Cao Y.-B. (2023). Experimental Investigations on Mechanical and Failure Behaviors of Transversely Isotropic Shale Containing Twin Fissures under True Triaxial Stresses. Arch. Appl. Mech..

[29] Zhang Y., Jing W., Jing L., Jin R., Cheng P. (2023). Numerical simulation of the effects of fissure inclination and length on rock strength and damage characteristics. Coal Technol..

[30] Wang Y., Tang J., Dai Z., Yi T. (2018). Experimental Study on Mechanical Properties and Failure Modes of Low-Strength Rock Samples Containing Different Fissures under Uniaxial Compression. Eng. Fract. Mech..

[31] Li S.-C., Kang Y.-L., Luo P.-Y. (2009). Effect of stress on fracture width and permeability of coal rock. Coalf. Geol. Explor..

